# Simultaneous removal of methylene blue and Cd(II) by pecan shell–based activated carbon: adsorption kinetics, isotherms, and physicochemical characterization

**DOI:** 10.1186/s40643-026-01117-1

**Published:** 2026-08-17

**Authors:** Jae-Hyeok Lee, Jae-Wook Lee, Sung-Hee Roh

**Affiliations:** 1 College of General Education, Chosun University, 309 Pilmoon-daero, Dong-gu, Gwangju, 61452 Republic of Korea; 2 Department of Chemical and Biochemical Engineering, Chosun University, 309 Pilmoon-daero, Dong-gu, Gwangju, 61452 Republic of Korea

**Keywords:** Pecan shell–based activated carbon, Simultaneous removal, Methylene blue, Cd(II), Binary adsorption system

## Abstract

The coexistence of organic dyes and heavy metals in wastewater requires efficient and sustainable adsorbents capable of simultaneous contaminant removal. In this study, pecan shell–based activated carbons (PSC-0–PSC-3) were prepared by KOH activation using different impregnation ratios; their physicochemical properties and adsorption performance toward methylene blue (MB) and Cd(II) were systematically investigated under single- and binary-component systems. Among the prepared adsorbents, PSC-3 exhibited the highest BET specific surface area (2857 m^2^ g^−1^) and total pore volume (1.61 cm^3^ g^−1^). MB adsorption was best described by the Freundlich isotherm model (R^2^ = 0.998), indicating heterogeneous adsorption behavior. The Langmuir model estimated a maximum adsorption capacity of 529.5 mg g^−1^. Under binary-component conditions, PSC-3 achieved nearly complete MB removal (≈ 100%), whereas Cd(II) removal was strongly dependent on solution pH and adsorbent dosage. The adsorption of Cd(II) was primarily governed by electrostatic attraction and surface complexation with oxygen-containing functional groups. Adsorption–desorption experiments demonstrated that PSC-3 retained approximately 85% and 80% of its initial adsorption efficiency for MB and Cd(II), respectively, after five regeneration cycles, indicating good reusability. These findings demonstrate that PSC-3 is a low-cost, sustainable, and highly efficient adsorbent with strong potential for the simultaneous removal of organic dyes and heavy metal ions from mixed wastewater.

**Graphical abstract:**

**Figure:**
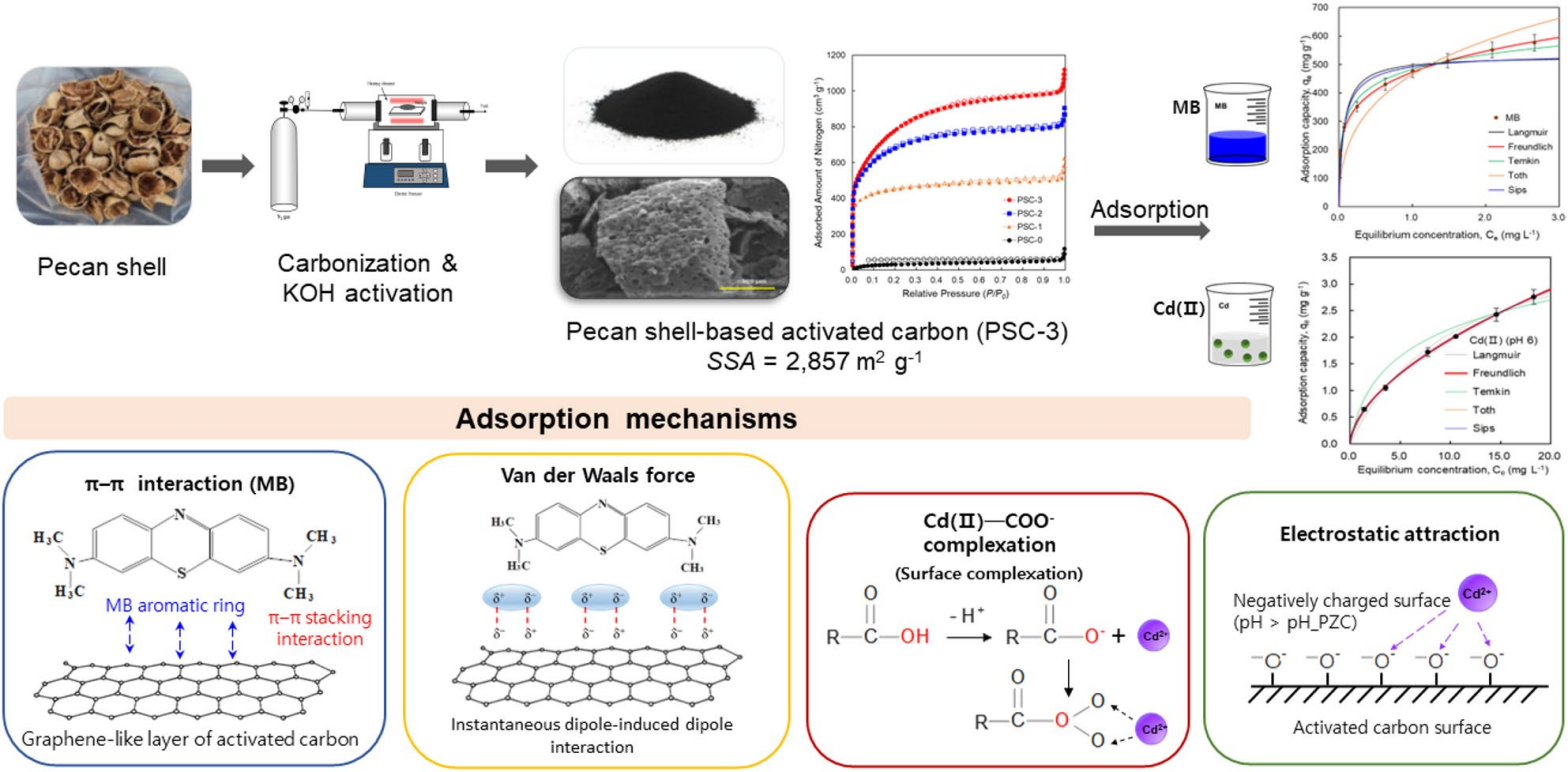

**Supplementary Information:**

The online version contains supplementary material available at 10.1186/s40643-026-01117-1.

## Introduction

Water pollution, exacerbated by rapid industrialization and agricultural activities, remains a major global environmental issue. Despite considerable advancements in pollution mitigation and environmental protection legislation, dye residues and heavy metals are still frequently detected in wastewater from various industries, such as the textile, paper, leather, paint, cosmetics, pesticide, and pharmaceutical industries (Alkan et al. 2004; Turhan and Ozturkcan 2013). Even at extremely low concentrations, dye residues diminish the aesthetic value of wastewater treatment effluent; if discharged without proper treatment, this effluent can cause several environmental issues in water systems, including reduced light transmittance, the inhibition of photosynthesis, and increased chemical oxygen demand (Métivier-Pignon et al. 2003; Owamah et al. 2013; Alharby et al. 2021). Additionally, methylene blue (MB), a synthetic dye, is highly toxic at elevated concentrations and causes skin irritation, vomiting, meningitis, and cancer (Jawad et al. 2018; Lellis et al. 2019; Shooto et al. 2020; Thabede et al. 2020; Alsafran et al. 2023).

Heavy metals, owing to their toxicity, bioaccumulation potential, and persistence in the environment, also constitute a major environmental concern. Notably, cadmium is a highly toxic metal; because of its adverse health effects, it is listed among the WHO's “Top 10 Chemicals of Critical Public Health Concern” (Hasan et al. 2019; Patel 2020; Zhu et al. 2020; Angaru et al. 2022). Furthermore, given its non-degradable nature, it easily accumulates in living organisms and environmental matrices, such as water and soil. Even at low concentrations, it can lead to serious health complications, including brain damage, chronic kidney disease, lung disease, and skeletal damage (Jawad et al. 2018; Lellis et al. 2019; Shooto et al. 2020; Thabede et al. 2020; Alsafran et al. 2023).

Over the years, adsorption has attracted considerable attention as an effective strategy for removing contaminants from aquatic environments. In this regard, activated carbon has been extensively used in water treatment processes owing to its advantages, including a porous structure, high specific surface area (SSA), and the presence of various modifiable functional groups on its surface. However, the high manufacturing cost and reliance on non-renewable resources pose sustainability challenges (Bello et al. 2022; Jia et al. 2022; Jawad et al. 2022a; Liu et al. 2023). To address these issues, active research has been conducted to produce activated carbon from lignocellulosic biomass residues such as pecan shells, almond shells, coconut shells, fruit seeds (e.g., jujube seeds), and wood. As precursors to granular activated carbon, shells are advantageous because of their low cost, renewability, environmentally friendly nature, low ash content, and high adsorption capacities (Heschel and Klose 1995; Toles et al. 1997; Johns et al. 1998). Reportedly, pecan shell–based activated carbon (PSBAC) demonstrates excellent adsorption characteristics, with its SSA and total pore volume reaching 1100 m^2^ g^−1^ and 0.5–0.6 cm^3^ g^−1^, respectively (Dastgheib and Rockstraw 2001; Kaveeshwar et al. 2018a; Kaveeshwar et al. 2018b). Thus, PSBAC is a promising adsorbent for the removal of various contaminants from wastewater.

In real wastewater environments, organic pollutants and heavy metals are generally present as complex mixtures rather than as isolated contaminants. Accordingly, developing technologies that can effectively remove these two contaminant classes concurrently is important. Utilizing a single adsorbent to target multiple pollutants can streamline the treatment train, enhance process efficiency, and lower operating costs.

Recent studies have reported the development of biomass-derived activated carbons for the removal of methylene blue (MB) and heavy metal ions from aqueous solutions. Biomass-based activated carbons have demonstrated excellent adsorption capacities toward MB or Cd(II) owing to their well-developed porous structures and abundant surface functional groups (Lima et al. 2019; Khurshid et al. 2025; Frescura et al. 2026). However, most previous studies have focused on the adsorption of a single pollutant under single-component systems, evaluating the removal of either dyes or heavy metal ions separately. Consequently, the adsorption behavior and competitive interactions in mixed-contaminant systems remain insufficiently understood.

In practical wastewater, organic dyes and heavy metal ions frequently coexist and may compete for the available adsorption sites, resulting in adsorption characteristics that differ significantly from those observed in single-component systems. Therefore, the simultaneous removal of organic dyes and heavy metal ions using a single adsorbent remains an important challenge for practical wastewater treatment.

In this study, a high-performance pecan shell–based activated carbon (PSC-3) was synthesized from a lignocellulosic agricultural by-product through KOH activation, producing an adsorbent with an exceptionally high specific surface area (2857 m^2^ g^−1^) and abundant surface functional groups. Unlike previous studies on pecan shell–derived activated carbons, the present work systematically investigates the simultaneous removal of MB and Cd(II) in a binary-component system, including adsorption kinetics, equilibrium isotherms, pH-dependent adsorption behavior, competitive adsorption, and adsorption mechanisms. Furthermore, the regeneration performance and cost-effectiveness of PSC-3 were evaluated to assess its practical applicability. These findings provide new insights into the application of pecan shell–derived activated carbon for treating mixed wastewater containing organic dyes and heavy metal ions and demonstrate its potential for practical wastewater remediation.

## Materials and methods

### Materials

Pecan shells were obtained from a pecan farm located in Sunchang-gun, Jeonbuk State, Republic of Korea. KOH, HCl, and MB were purchased from Daejung Chemical (Siheung, Korea), whereas cadmium was obtained from Kanto Chemical Co., Inc. (Tokyo, Japan). All reagents were used as received, without further purification.

### Synthesis of PSBAC

**Fig. 1:**
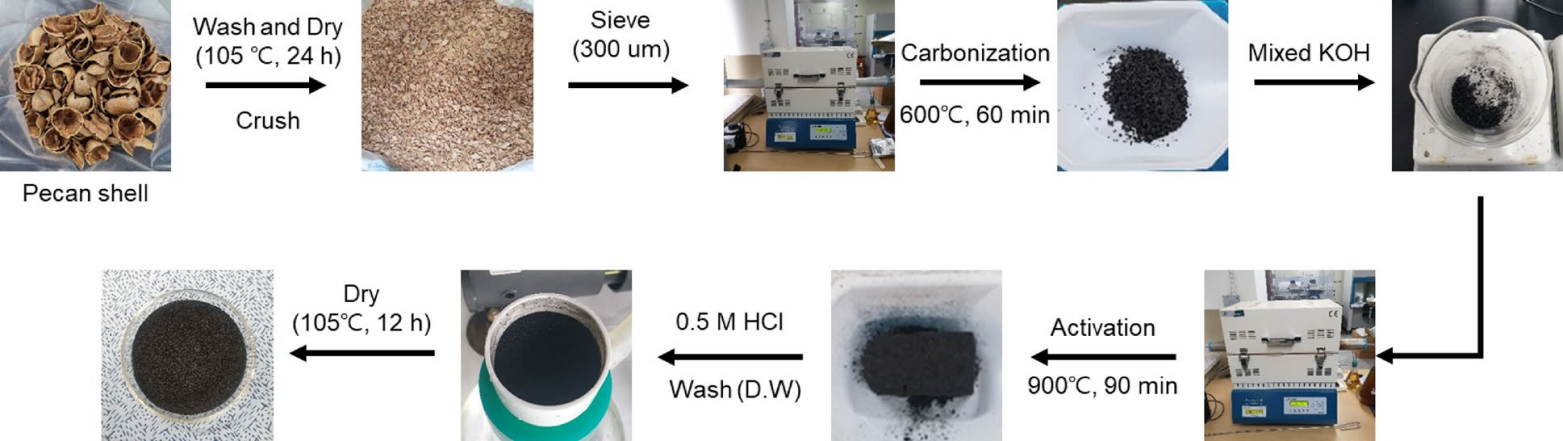
Schematic representation of the production process of pecan shell–based activated carbon (PSBAC)

A schematic representation of the PSBAC manufacturing process is provided in Fig. 1. In brief, pecan shells were washed thoroughly with distilled water to remove surface impurities and subsequently dried in a hot-air oven at 105 °C for 24 h. Thereafter, the washed and dried pecan shells were ground using a blender and sieved to obtain particles with sizes ≤ 300 μm.

For each carbonization batch, 35 g of the dried pecan shell powder was used. Next, the particles were carbonized in a tubular furnace under an N₂ atmosphere at a heating rate of 5 °C min^−1^ to 600 °C; they were maintained at this temperature for 60 min. Thereafter, the carbonized material was mixed with KOH at mass ratios of 1:1, 1:2, and 1:3 (w/w), followed by the addition of 10 mL of distilled water and stirring at 120 °C for 3 h.

After drying, the resulting products were activated in a tubular furnace (FU 80-STG, Samheung Energy, Sejong, Korea) under an N_2_ atmosphere at a heating rate of 5 °C min^−1^ to 900 °C, which was held for 90 min. Finally, the activation products were washed with a 0.5 M HCl solution and then with distilled water until a neutral pH was attained, filtered, and dried in a dry oven at 105 °C for 12 h to obtain PSBAC. The carbonized pecan shells without KOH activation were designated PSC-0, whereas those treated with KOH at pecan shell-to-KOH mass ratios of 1:1, 1:2, and 1:3 were designated PSC-1, PSC-2, and PSC-3, respectively.

### Characterization of pecan shell–based adsorbents

The physicochemical characteristics of the pecan shell–based adsorbents with and without KOH treatment, i.e., surface morphology, surface functional groups, SSA, and pore size distribution, were analyzed. In brief, surface characteristics were observed using field-emission scanning electron microscopy (FE-SEM) at an acceleration voltage of 15 kV with an SU-8600 Hitachi system (Tokyo, Japan). The elemental compositions of the activated carbons were analyzed using energy-dispersive X-ray spectroscopy (EDX) attached to the FE-SEM system (SU-8600, Hitachi, Tokyo, Japan). The weight percentages of carbon (C), oxygen (O), and potassium (K) were determined from the acquired spectra to evaluate the compositional changes resulting from KOH activation. For surface functional group analysis, FT-IR spectroscopy was performed using the Spectrum 3 system (PerkinElmer, Norwalk, CT, USA). Further, N_2_ adsorption–desorption measurements were performed using the nanoPOROSITY-XQ system (Mirae Scientific Instruments Inc., Daejeon, Korea) to determine the SSA and pore structure. Prior to these analyses, all the samples were degassed at 300 °C for 360 min to remove moisture and adsorbed gases. Thereafter, SSA was determined based on N_2_ desorption isotherms using the Brunauer–Emmett–Teller (BET) method, whereas pore size distribution was determined using the Horváth–Kawazoe model (Li et al. 2020).

The pH at the point of zero charge (pH_PZC) of PSC-3 was determined using the pH-drift method. Briefly, 50 mL of 0.01 M NaCl solution was adjusted to initial pH values ranging from 2 to 12 using 0.1 M HCl or 0.1 M NaOH. Subsequently, 0.05 g of PSC-3 was added to each solution; the suspensions were agitated at 25 °C for 24 h. The final pH values were measured; pH_PZC was determined from the intersection point where the initial pH equaled the final pH (pH_i_ = pH_f_).

### Adsorption experiments

MB and Cd(II) adsorption experiments were performed using the pecan shell–based activated carbon with the optimal structural properties (PSC-3). Working solutions were prepared by diluting 1000 mg L^−1^ MB and Cd(II) standard solutions with deionized water to the desired concentrations.

For MB adsorption kinetics, a 100 mg L^−1^ MB solution was mixed with 0.10 g of PSC-3 in 0.50 L of solution and agitated in an orbital shaker (180 rpm) at 25 °C for 360 min. For MB adsorption isotherm experiments, MB solutions with initial concentrations of 20–60 mg L^−1^ were mixed with 0.025 g of PSC-3 in 0.25 L of solution and agitated in an orbital shaker (180 rpm) at 25 °C until equilibrium (720 min).

For Cd(II) adsorption kinetics, a 2.0 mg L^−1^ Cd(II) solution was mixed with 0.15 g of PSC-3 in 0.25 L of solution and agitated in an orbital shaker (180 rpm) at pH 6.0 and 25 °C for 1440 min. For Cd(II) adsorption isotherm experiments, Cd(II) solutions with initial concentrations of 2–20 mg L^−1^ were mixed with 0.15 g of PSC-3 in 0.25 L of solution and agitated in an orbital shaker (180 rpm) at pH 3.0, 4.0, and 6.0 at 25 °C until equilibrium (1440 min). The solution pH was adjusted using 0.1 M HCl and 0.1 M NaOH (Daejung Chemical, Siheung, Korea).

After adsorption, the suspensions were filtered through a 0.45 μm PTFE membrane filter. The residual concentration of methylene blue (MB) was determined using a UV–Vis spectrophotometer (Ultrospec 2100 Pro, Amersham Biosciences) at the maximum absorbance wavelength of MB (λmax = 660 nm). The residual concentration of Cd(II) was analyzed using inductively coupled plasma mass spectrometry (ICP-MS; iCAP RQplus ICP-MS, Thermo Fisher Scientific). The removal efficiency (%) and adsorption capacity (*q*_*e*_) were calculated based on the initial and residual concentrations of the adsorbates.

All adsorption experiments were performed in triplicate (*n* = 3). Data are presented as the mean of three independent experiments; the error bars represent the experimental variability.

### Simultaneous removal of MB and Cd(II) in a binary system

Simultaneous removal experiments for MB and Cd(II) were conducted in a binary system using the pecan shell–based activated carbon with the optimal structural properties (PSC-3). Binary working solutions were prepared by mixing appropriate volumes of 1000 mg L^−1^ methylene blue (MB) and Cd(II) stock solutions and diluting the mixture with deionized water to a final volume of 250 mL, resulting in initial concentrations of 50 mg L^−1^ MB and 2.0 mg L^−1^ Cd(II). The solution was thoroughly mixed prior to the adsorption experiments to ensure homogeneity.

The initial concentrations of MB and Cd(II) were fixed at 50 mg L^−1^ and 2.0 mg L^−1^, respectively. The MB concentration was selected because it is widely used in adsorption studies and allows the reliable evaluation of dye adsorption performance. The Cd(II) concentration was selected to represent contaminated wastewater conditions while ensuring accurate quantification and assessment of adsorption behavior. These concentrations were selected to evaluate the simultaneous removal performance of PSC-3 under controlled laboratory conditions rather than to replicate a specific wastewater composition.

To evaluate the effects of solution pH and adsorbent dosage on simultaneous removal performance, the pH of the binary solution was adjusted to 3, 7, or 10 using 0.1 M HCl and 0.1 M NaOH (Daejung Chemical, Siheung, Korea). The PSC-3 dosage was varied from 2 to 5 g L^−1^. The adsorption experiments were carried out at 25 °C for 30 min. After adsorption, the suspensions were filtered through a 0.45 μm PTFE membrane filter; the residual MB concentration was determined using a UV–Vis spectrophotometer (Ultrospec 2100 Pro, Amersham Biosciences) at the maximum absorbance wavelength of MB (λ_max_ = 660 nm). The residual Cd(II) concentration was analyzed using ICP-MS (iCAP RQplus ICP-MS, Thermo Fisher Scientific). All binary adsorption experiments were also performed in triplicate (*n* = 3). The results are presented as the mean of three independent experiments.

## Results and discussion

### Surface morphology and elemental composition of pecan shell–based adsorbents

**Fig. 2:**
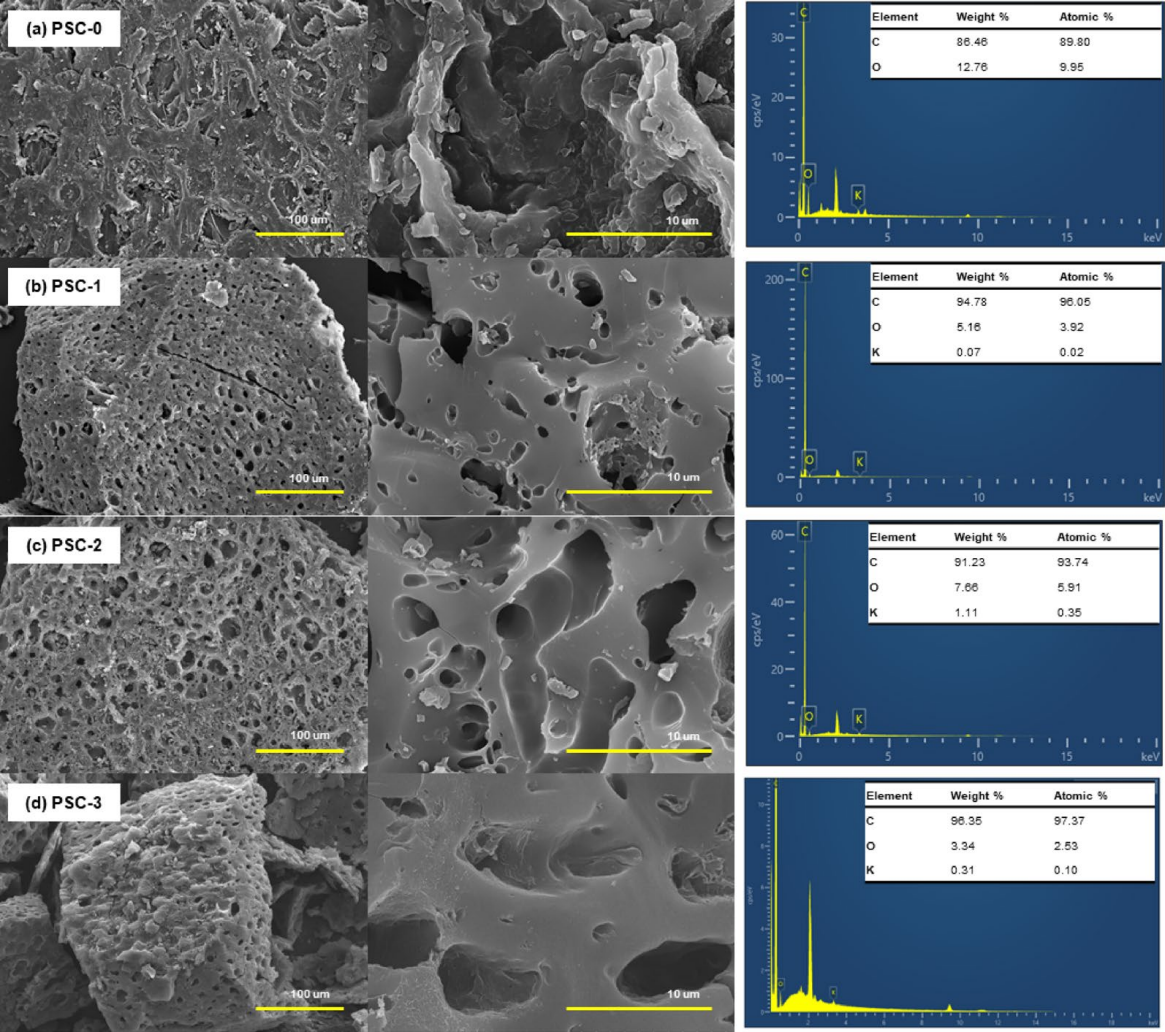
SEM images and EDX spectra of pecan shell–based activated carbons. **a** PSC-0, **b** PSC-1, **c** PSC-2, and **d** PSC-3. PSC-0 represents the carbonized pecan shell without KOH treatment, whereas PSC-1–PSC-3 are KOH-activated samples prepared at pecan shell-to-KOH mass ratios of 1:1, 1:2, and 1:3, respectively

The surface properties and chemical compositions of the pecan shell–based adsorbents investigated in this study (PSC-0–PSC-3) are presented in Fig. 2. The SEM image of carbonized pecan shells without KOH treatment (PSC-0) showed an irregular surface morphology without any discernible pore structure (Fig. 2a). In contrast, the SEM images of the KOH-treated samples (PSC-1–PSC-3) showed well-developed pore structures, indicating that chemical activation using KOH markedly enhanced porosity (Fig. 2b–d). Moreover, pore size increased as the KOH mass ratio increased, suggesting that at elevated concentrations, the activating agent facilitated mesopore development.

EDX analysis further confirmed changes in the elemental composition of the pecan shell–based activated carbons after KOH activation. The carbon and oxygen contents of PSC-0 were 86.46 and 12.76 wt%, respectively. After KOH activation, the carbon contents of PSC-1, PSC-2, and PSC-3 were 94.78, 91.23, and 96.35 wt%, respectively, whereas their oxygen contents were 5.16, 7.66, and 3.34 wt%, respectively.

The relatively high oxygen content observed for PSC-2 may be attributed to the partial formation and retention of oxygen-containing surface functionalities during intermediate KOH activation. At this stage, oxidation and pore-forming reactions can generate oxygenated groups on the carbon surface. However, at the relatively high KOH ratio used for PSC-3, extensive carbonization, aromatization, and deoxygenation occurred, resulting in a relatively low oxygen content and a carbon-rich structure.

Additionally, the lower K content in PSC-3 (0.31 wt%) than in PSC-2 (1.11 wt%) is likely due to the more extensive activation and subsequent acid-washing process. During high-temperature KOH activation, potassium-containing species participate in pore formation and are then effectively removed during HCl washing and repeated washing with deionized water. Consequently, PSC-3 retained less residual potassium than PSC-2. These results support the conclusion that relatively high KOH loading enhances pore development and produces a relatively carbon-rich activated carbon surface.

### SSA and pore structure of pecan shell–based adsorbents

**Fig. 3:**
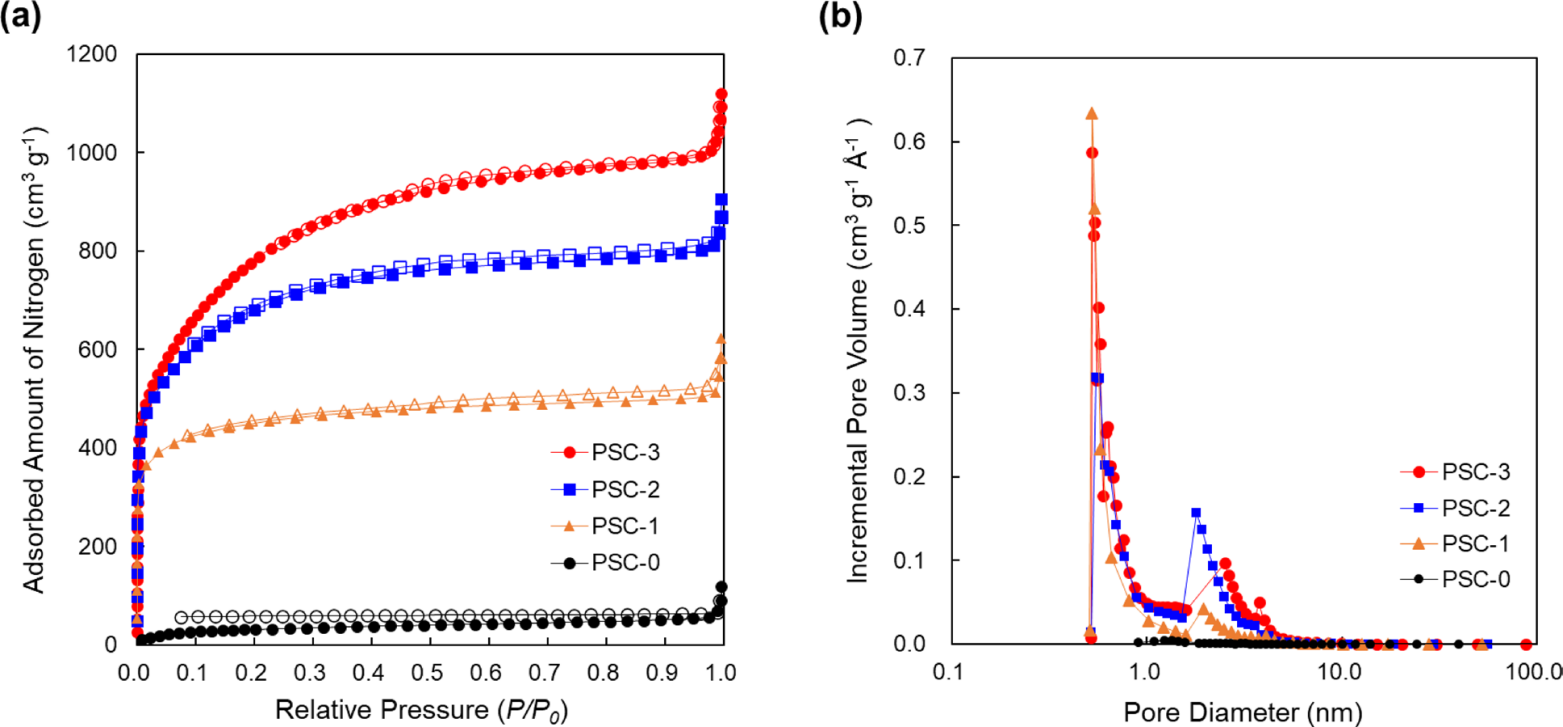
**a** N_2_ adsorption isotherms and **b** pore size distributions of pecan shell–based activated carbons. PSC-0 represents the carbonized pecan shell without KOH treatment, whereas PSC-1–PSC-3 are KOH-activated samples prepared at pecan shell-to-KOH mass ratios of 1:1, 1:2, and 1:3, respectively

The adsorption properties of activated carbon are closely associated with its SSA (Danish and Ahmad 2018; Mojoudi et al. 2019; Lan et al. 2020). Figure 3a shows the N_2_ adsorption–desorption isotherms of the pecan shell–based adsorbents. For PSC-0, nitrogen adsorption reached saturation at low relative pressures, indicating limited pore development. Conversely, PSC-1–PSC-3 exhibited Type I isotherms (IUPAC classification), implying a predominantly microporous structure; as the KOH mass ratio increased, the amount of nitrogen adsorbed in the low-pressure region also increased, indicating enhanced microporosity. Furthermore, PSC-1–PSC-3 exhibited hysteresis loops in the relative pressure range of 0.4–0.9, suggesting the presence of mesopores. Figure 3b shows the pore-size distribution characteristics of each sample. For PSC-0, pores were virtually nonexistent, whereas PSC-1–PSC-3 predominantly contained small micropores with sizes of 0.7–1.5 nm. As the KOH mass ratio increased, the pore size distribution showed a decrease in micropore density and an increase in mesopore density, suggesting that KOH activation promoted mesopore formation.

Table 1 shows the BET characteristics of each sample. PSC-0 showed a low SSA of 119 m^2^ g^−1^ and a pore volume of 0.06 cm^3^ g^−1^, whereas PSC-1–PSC-3 showed significantly higher SSAs, micropore surface areas, and mesopore surface areas in the ranges of 1,552–2,857, 1,245–1,735, and 344–631 m^2^ g^−1^, respectively. The micropore and mesopore volumes of PSC-1–PSC-3 also increased from 0.65 to 1.01 cm^3^ g^−1^ and from 0.29 to 0.60 cm^3^ g^−1^, respectively. Additionally, the average pore sizes of PSC-1–PSC-3 were 2.4, 2.7, and 2.3 nm, respectively.

**Table 1:** BET parameters of pecan shell–based adsorbents at different KOH mass ratios

Samples	*S*_*BET*_^a^ (m^2^ g^−1^)	*S*_*meso*_^b^ (m^2^ g^−1^)	*S*_*mic*_^c^ (m^2^ g^−1^)	*V*_*Total*_^d^ (cm^3^ g^−1^)	*V*_*meso*_^e^ (cm^3^ g^−1^)	*V*_*mic*_^f^ (cm^3^ g^−1^)	*D*_*a*_^g^ (nm)
PSC-0	119.62	16.23	47.29	0.06	0.02	0.04	2.01
PSC-1	1552.43	344.28	1245.38	0.94	0.29	0.65	2.42
PSC-2	2451.77	1155.46	1610.82	1.64	0.73	0.91	2.68
PSC-3	2857.47	631.17	1735.39	1.61	0.60	1.01	2.25

^a^Specific surface area determined using the BET method

^b^Mesopore surface area

^c^Micropore surface area

^d^Total pore volume

^e^Mesopore volume

^f^Micropore volume

^g^Average pore diameter $\left( {D_{a} = \frac{{4 \times V_{Total} \times 1000}}{{S_{BET} }}} \right)$

The exceptionally high BET specific surface area of PSC-3 (2857 m^2^ g^−1^) is attributed to the highly effective KOH activation process. During chemical activation, KOH promotes carbon etching, pore generation, and pore widening, resulting in a well-developed hierarchical porous structure containing abundant micropores and mesopores. The large specific surface area provides numerous accessible adsorption sites and facilitates the rapid diffusion of adsorbates, thereby contributing to the excellent adsorption performance of PSC-3 toward MB and Cd(II).

These findings confirmed that the KOH mass ratio significantly influenced the SSA of the pecan shell–based adsorbents and that increasing the KOH mass ratio enhanced the formation of mesopores as micropores collapsed. Therefore, owing to the optimal structural properties of PSC-3, it was selected for subsequent adsorption kinetics and isotherm studies.

### Surface functional groups in pecan shell–based adsorbents

**Fig. 4:**
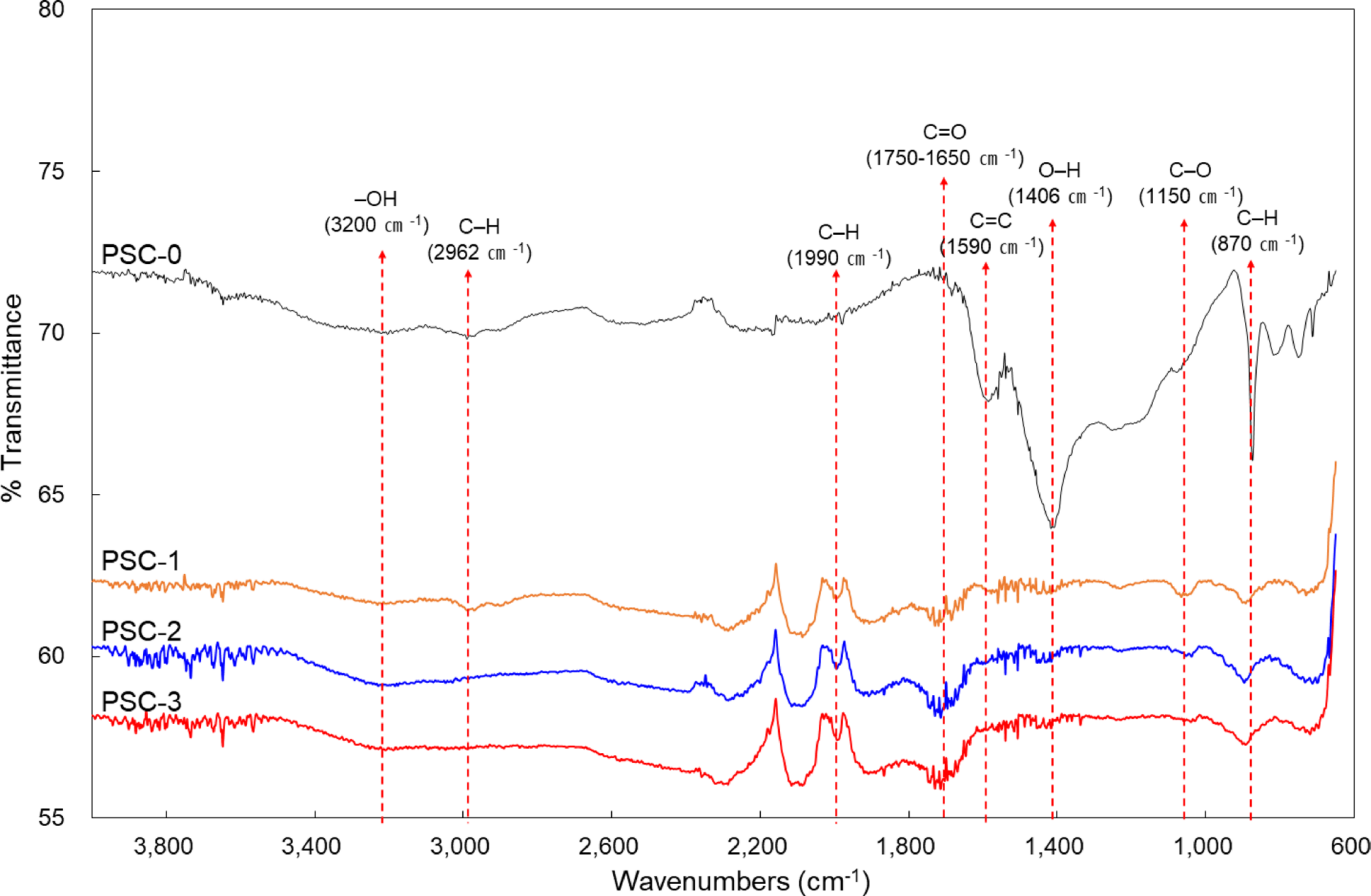
Fourier transform infrared (FT-IR) spectra of pecan shell–based activated carbons prepared at different KOH mass ratios. PSC-0 represents the carbonized pecan shell without KOH treatment, whereas PSC-1–PSC-3 are KOH-activated samples prepared at pecan shell-to-KOH mass ratios of 1:1, 1:2, and 1:3, respectively

The results of FT-IR spectroscopy used to identify the surface functional groups of PSC-0–PSC-3 are shown in Fig. 4. The absorption peak at 2962 cm^−1^ corresponded to the C–H stretching vibration of –CH_2_ groups (Azmi et al. 2016). A weak absorption band was observed near 2100 cm^−1^. Because this spectral region is uncommon for activated carbons, the band was not assigned to a specific functional group; instead, it may be associated with weak combination bands or background/instrumental effects. The band near 1990 cm^−1^ was attributed to the C–H bending vibration of aromatic compounds; the peaks at 1750–1650 cm^−1^ were assigned to the C=C stretching of aromatic carboxyl groups or the C=O stretching of carbonyl groups (Ali et al. 2020; Teğin et al. 2024). These peaks became increasingly pronounced after KOH activation followed by acid treatment, implying enhanced carbonyl (C=O) functionality and C=C stretching of aromatic rings. The peak at 1590 cm^−1^, attributed to the C=C stretching vibration of the cyclic alkenes, showed decreased intensity after KOH activation. The peaks at 1406 cm^−1^ and 1100–1030 cm^−1^ were attributed to O–H bending and C–O stretching vibrations, respectively. Additionally, the broad and intense band between 1100 and 1043 cm^−1^ was attributed to the C–O stretching vibrations of primary and secondary R–OH groups in alcohols; the peaks below 1000 cm^−1^ were attributed to less reactive inorganic functional groups (Xiong et al. 2024). Overall, increasing the KOH-to-precursor mass ratio led to relatively pronounced oxygen-containing functional groups and an elevated degree of unsaturated carbon bonding (aromatization), suggesting that the adsorption behavior of the adsorbents was governed by the combined effects of surface oxygen functionalities and aromatic carbon structures.

### Adsorption characteristics of MB and Cd(II) on PSC-3

MB, a cationic dye that is extensively used in textile and dyeing processes, is often employed as a model compound in adsorption studies of organic contaminants. Its removal by activated carbon is primarily driven by adsorption within mesopores (Li et al. 2020). Therefore, MB was used in this study to evaluate the adsorption capacity and pore characteristics of PSC-3.

#### MB adsorption kinetics

To elucidate the adsorption mechanism of PSC-3, various adsorption kinetic models, namely the pseudo-first-order and pseudo-second-order, Weber–Morris intraparticle diffusion, Urano–Tachikawa, and S-shape models, were applied. Recently, the use of linearized kinetic models has become controversial because of incomplete data fitting and statistical bias (Bahrudin et al. 2019). Therefore, the non-linear forms of the five aforementioned adsorption models were used to estimate MB adsorption parameters based on experimental data. The estimations were performed using MATLAB R2023a (MathWorks Inc., Natick, MA, USA). Thereafter, the model with the highest coefficient of determination (R^2^) was considered the most appropriate adsorption kinetic model. The non-linear forms of the pseudo-first-order and pseudo-second-order models are shown in Eqs. (1) and (2), respectively (Bahrudin et al. 2019; Wang and Guo 2023).

1 $$ q_{t} = q_{e} \left( {1 - e^{{ - k_{1} t}} } \right) $$

2 $$ q_{t} = \frac{{k_{2} q_{e}^{2} t}}{{1 + k_{2} q_{e} t}} $$

where $q_{e}$ (mg g^−1^) and $q_{t}$ (mg g^−1^) represent the amounts of MB adsorbed by PSC-3 at equilibrium and at time *t*, respectively; $k_{1}$ (min^−1^) and $k_{2}$ (g mg^−1^ min^−1^) represent the pseudo-first-order and pseudo-second-order rate constants, respectively. Additionally, the Weber–Morris intraparticle diffusion, Urano–Tachikawa equation, and S-shape kinetic model, as shown in Eqs. (3)–(5), respectively, were used to characterize diffusion within the adsorption system (Wang and Guo 2023).

3 $$ q_{t} = k_{i} t^{1/2} + C $$

4 $$ q_{t} = q_{e} \left( {1 - e^{{ - 4\pi^{2} D_{i} t/d_{p}^{2} }} } \right)^{0.5} $$

5 $$ q_{t} = { }\frac{{q_{e} bt^{1/n} }}{{1 + bt^{1/n} }} $$

where $k_{i}$ (mg g^−1^ min^0.5^) and $D_{i}$ (m^2^ s^−1^) represent the intraparticle diffusion rate constant and intraparticle diffusion coefficient, respectively (Jawad et al. 2021); *b*(s^−1^) represents an adjustable parameter, and *n* in the S-shape kinetic model represents the adsorption/desorption ratio.

**Fig. 5:**
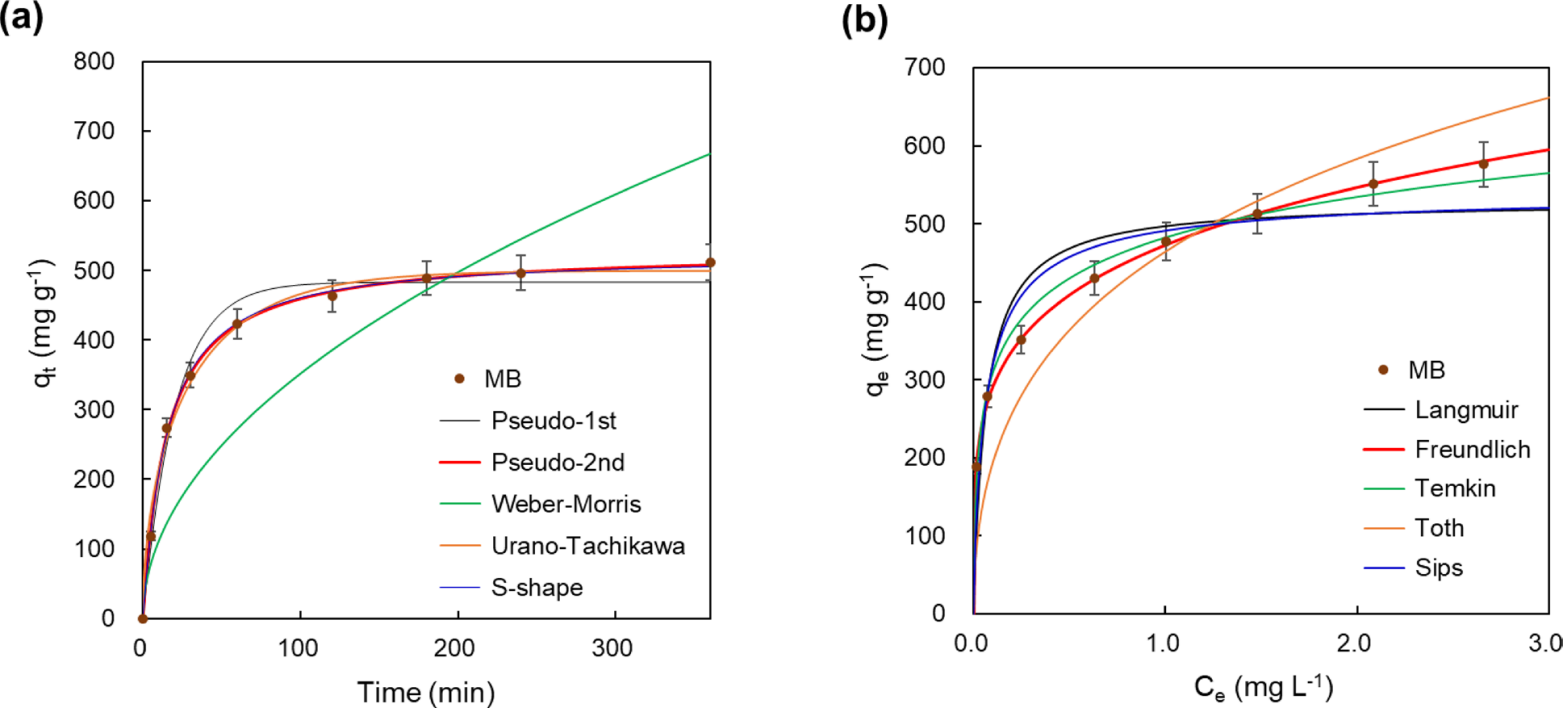
MB adsorption kinetics and isotherm model fits for PSC-3. **a** MB adsorption kinetics on PSC-3 and the corresponding kinetic model fits. **b** MB adsorption isotherms on PSC-3 and the corresponding isotherm model fits

Figure 5a shows the experimental MB adsorption kinetics and the corresponding fits to different kinetic models, whereas Table 2 summarizes the fit model parameters and R^2^ values. Specifically, the adsorption of MB on PSC-3 was best fit by the pseudo-second-order model (R^2^ = 0.999), as evidenced by the high degree of agreement between the calculated *q*_*e*_ values and the experimental values. This suggests that MB adsorption onto PSC-3 is primarily driven by chemical interactions between the active sites on the adsorbent surface and the cationic π electrons of MB.

**Table 2:** Adsorption kinetic parameters for methylene blue adsorption onto PSC-3

Model	Parameter	PSC-3
Pseudo-first-order	${q}_{e}$ (mg g^−1^)	483.2
	${k}_{1}$ (min^−1^)	0.048
	${R}^{2}$	0.986
Pseudo-second-order	${q}_{e}$ (mg g^−1^)	529.3
	${k}_{2}$ (g mg^−1^ min^−1^)	1.0 × 10^−^4
	${R}^{2}$	0.999
Weber–Morris	${K}_{i}$ (mg g^−1^ min^−0.5^)	35.1
	${R}^{2}$	0.620
Urano–Tachikawa	${q}_{e}$ (mg g^−1^)	499.6
	${D}_{i}$ (m^2^ s^−1^)	8.28 × 10^–11^
	${R}^{2}$	0.992
S-shape	${q}_{e}$ (mg g^−1^)	523.4
	*n*	0.9531
	*b* (min^−1^)	0.0587
	${R}^{2}$	0.998

Experimental conditions: initial methylene blue concentration (C₀) = 100 mg L^−1^, adsorbent dosage = 0.10 g in 0.50 L of solution, solution pH = 6.0, temperature = 25 °C, and contact time = 360 min

Additionally, although the pseudo-second-order model provided an excellent fit (R^2^ = 0.999), the relatively low coefficient of determination obtained for the Weber–Morris intraparticle diffusion model (R^2^ = 0.620) indicated that intraparticle diffusion was not the sole rate-controlling mechanism. This result suggested that boundary-layer (film) diffusion also contributed to the adsorption process. Therefore, MB adsorption onto PSC-3 was governed by multiple mass-transfer steps, including external diffusion, intraparticle diffusion, and surface adsorption. Similar multi-step adsorption behavior has been widely reported for porous activated carbon adsorbents (Weber and Morris 1963; Ho and McKay 1999).

#### MB adsorption isotherms

Adsorption isotherms provide valuable insights into adsorption mechanisms, pollutant concentrations in the liquid phase, and the equilibrium amounts of substances adsorbed on solid surfaces (Murphy et al. 2023). In this study, the interaction between MB and PSC-3 was evaluated using the Langmuir, Freundlich, Temkin, Toth, and Sips adsorption isotherms. The Langmuir isotherm describes monolayer adsorption on uniformly distributed adsorption sites. The Freundlich model describes multilayer adsorption at different surface energies. The Temkin model explains the decrease in heat of adsorption as surface adsorption coverage increases owing to interactions between adsorbent and adsorbate. Furthermore, the Toth isotherm is an empirical modification of the Langmuir model that effectively describes adsorption behavior on heterogeneous surfaces across a wide concentration range, whereas the Sips isotherm combines the Langmuir and Freundlich isotherms to effectively model adsorption processes on heterogeneous surfaces.

The non-linear equations for the Langmuir, Freundlich, Temkin, Toth, and Sips isotherms are provided in Eqs. (6)–(10), respectively (Bahrudin et al. 2019; Mabuza et al. 2022; Wang and Guo 2023).

6 $$ q_{e} = \frac{{q_{m} bC_{e} }}{{1 + bC_{e} }} $$

7 $$q_{e} = K_{F}C_{e}^{\frac{1}{n_{F}}}$$

8 $$ q_{e} = B ln\left( {K_{T} C_{e} } \right) $$

9 $$ q_{e} = \frac{{q_{m} K_{T} C_{e} }}{{\left[ {1 + \left( {K_{T} C_{e} } \right)^{{n_{T} }} } \right]^{{\frac{1}{{n_{T} }}}} }} $$

10 $$ q_{e} = \frac{{q_{m} K_{S} C_{e}^{{n_{s} }} }}{{1 + K_{S} C_{e}^{{n_{s} }} }} $$

In the Langmuir model (Eq. 6), $q_{m}$ (mg g^−1^) represents the maximum adsorption capacity for MB, $C_{e}$ (mg L^−1^) represents the equilibrium MB concentration, and *b* (L mg^−1^) is the Langmuir constant representing adsorption affinity. In the Freundlich isotherm (Eq. 7), $K_{F}$ (L mg^−1^) and $n_{F}$ are parameters associated with adsorption capacity and adsorption intensity, respectively; in the Temkin model (Eq. 8), *B* is a constant related to the heat of adsorption, and $K_{T}$ (L mg^−1^) is a constant representing adsorption affinity and heterogeneity. In the Toth model (Eq. 9), $K_{T}$ (L mg^−1^) and $n_{T}$ are constants related to adsorption affinity and heterogeneity, respectively; in the Sips model (Eq. 10), *K*_*s*_ (L mg^−1^) and *n*_*s*_ are constants related to adsorption capacity and heterogeneity, respectively. The calculated model parameters are shown in Table 3, whereas a schematic representation of the different isotherm models is provided in Fig. 5b. From Table 3, it is evident that the Freundlich model showed the highest R^2^, implying that MB adsorption on PSC-3 primarily occurred via multilayer adsorption. The adsorption characteristics described by the Freundlich model can be evaluated based on *K*_*F*_ and *n* values. Specifically, $n_{F} < 1$ indicates weak interactions and, consequently, limited adsorption, whereas $n_{F} > 1$ indicates strong interactions and, consequently, favorable adsorption. The $n_{F}$ value for PSC-3 was 4.773 (> 1), indicating that PSC-3 was an effective adsorbent for MB.

**Table 3:** Adsorption isotherm parameters for methylene blue adsorption onto PSC-3

Model	Parameter	PSC-3
Langmuir	${q}_{m}$ (mg g^−1^)	529.50
	*b* (L mg^−1^)	15.424
	${R}^{2}$	0.848
Freundlich	${K}_{F}$ (L mg^−1^)	472.69
	${n}_{F}$	4.773
	${R}^{2}$	0.998
Temkin	*B*	75.39
	${K}_{T}$ (L mg^−1^)	604.229
	${R}^{2}$	0.984
Toth	${q}_{m}$ (mg g^−1^)	7628.57
	${n}_{T}$	0.148
	${K}_{T}$(L mg^−1^)	85.315
	${R}^{2}$	0.727
Sips	${q}_{m}$ (mg g^−1^)	542.72
	${n}_{S}$	1.206
	${K}_{S}$ (L mg^−1^)	9.596
	${R}^{2}$	0.896

Experimental conditions: initial methylene blue concentration (C₀) = 20–60 mg L^−1^, adsorbent dosage = 0.025 g in 0.25 L of solution, solution pH = 6.0, temperature = 25 °C, and equilibrium contact time = 720 min

In the Sips model, $n_{s}$ reflects the degree of deviation from a homogeneous adsorption surface. An $n_{s} = 1$ indicates that the Sips model simplifies to the Langmuir model, resulting in monolayer adsorption on a homogeneous surface, whereas $n_{s} \ne 1$ indicates a heterogeneous surface, with the model exhibiting behavior similar to that of the Freundlich isotherm at low adsorbate concentrations. For PSC-3, $n_{s}$ was determined to be 1.206. This observation suggested a slightly heterogeneous surface with cooperative adsorption behavior and indicated that MB molecules were adsorbed onto active sites exhibiting quasi-homogeneous characteristics. These findings are broadly consistent with the Freundlich-based interpretation, which supports adsorption on energetically heterogeneous surface sites of PSC-3 with possible non-ideal or multilayer adsorption behavior.

#### Cd(II) adsorption kinetics

**Fig. 6:**
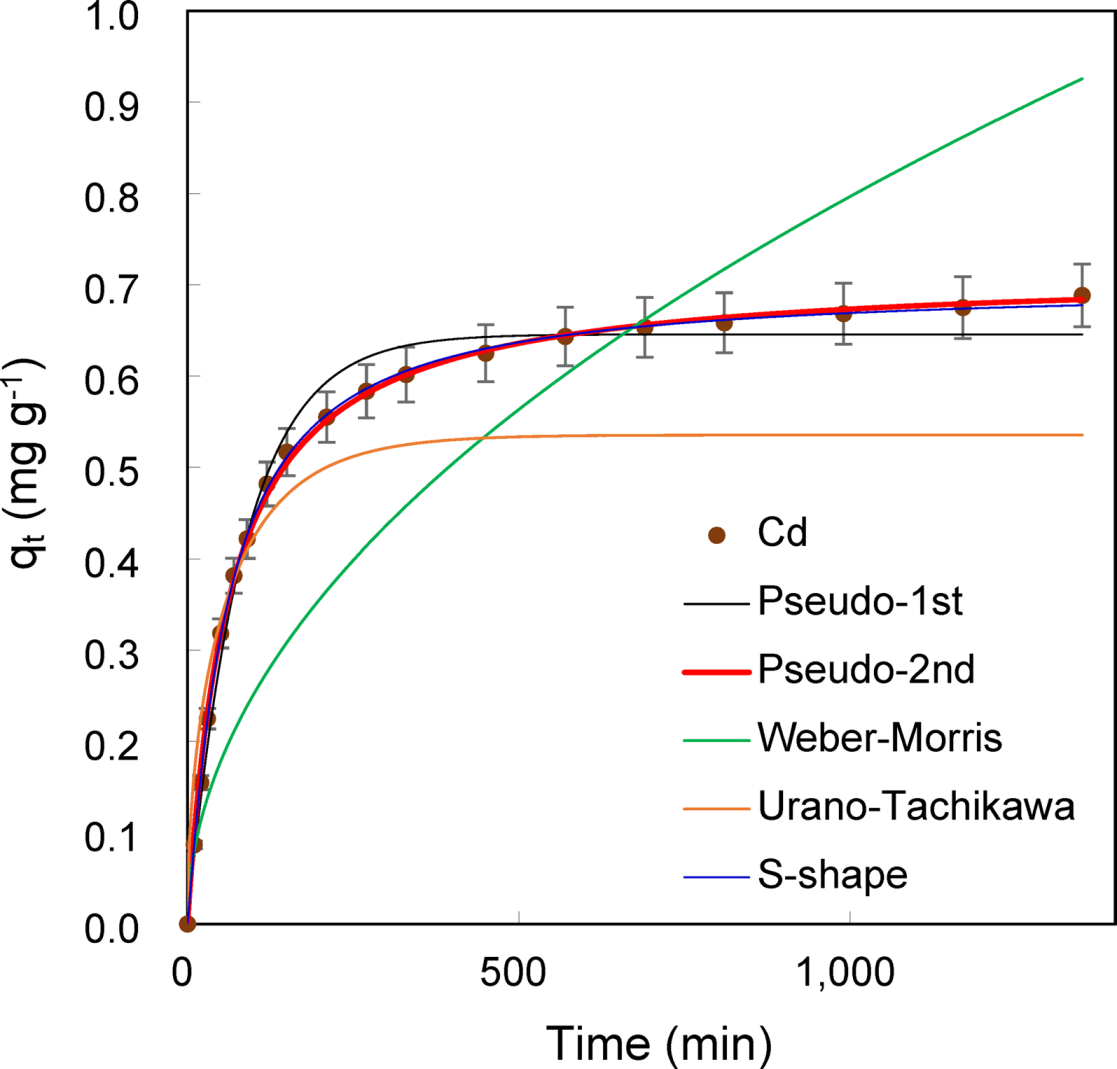
Cd(II) adsorption kinetics on PSC-3 and the corresponding kinetic model fits

The adsorption kinetics of Cd(II) onto PSC-3 were investigated to elucidate the adsorption behavior and possible rate-controlling mechanisms. As shown in Fig. 6, Cd(II) adsorption increased rapidly during the initial stage because of the abundance of available adsorption sites and gradually approached equilibrium after approximately 600 min. The experimental data were fit using the pseudo-first-order, pseudo-second-order, Weber–Morris intraparticle diffusion, Urano–Tachikawa, and S-shape kinetic models. The corresponding kinetic parameters are summarized in Table 4.

**Table 4:** Adsorption kinetic parameters for Cd(II) adsorption onto PSC-3

Model	Parameter	PSC-3
Pseudo-first-order	$${q}_{e} $$(mg g^−1^)	0.65
	${k}_{1}$ (min^−1^)	0.012
	${R}^{2}$	0.988
Pseudo-second-order	${q}_{e}$ (mg g^−1^)	0.72
	${k}_{2}$ (g mg^−1^ min^−1^)	0.022
	${R}^{2}$	0.998
Weber–Morris	${K}_{i}$ (mg g^−1^ min^−0.5^)	0.03
	${R}^{2}$	0.551
Urano–Tachikawa	${q}_{e}$ (mg g^−1^)	0.54
	${D}_{i}$ (m^2^ s^−1^)	2.237 × 10^–13^
	${R}^{2}$	0.835
S-shape	${q}_{e}$ (mg g^−1^)	0.70
	*n*	0.918
	*b* (min^−1^)	0.012
	${R}^{2}$	0.999

Experimental conditions: initial Cd(II) concentration (C₀) = 2.0 mg L^−1^, adsorbent dosage = 0.15 g in 0.25 L of solution, solution pH = 6.0, temperature = 25 °C, and contact time = 1440 min

Among the evaluated models, the S-shape kinetic model provided the best fit to the experimental data (R^2^ = 0.999), followed closely by the pseudo-second-order model (R^2^ = 0.998), indicating that the adsorption process was well described by a sigmoidal adsorption behavior with a rapid initial uptake followed by gradual saturation. In contrast, the relatively low coefficient of determination for the Weber–Morris model (R^2^ = 0.551) indicated that intraparticle diffusion alone was insufficient to describe the adsorption process, suggesting that boundary-layer diffusion also contributed to the overall adsorption kinetics. Overall, PSC-3 exhibited rapid Cd(II) adsorption and reached near-equilibrium within approximately 600 min, demonstrating its effectiveness as an adsorbent for Cd(II) removal.

#### Cd(II) adsorption isotherms

**Fig. 7:**
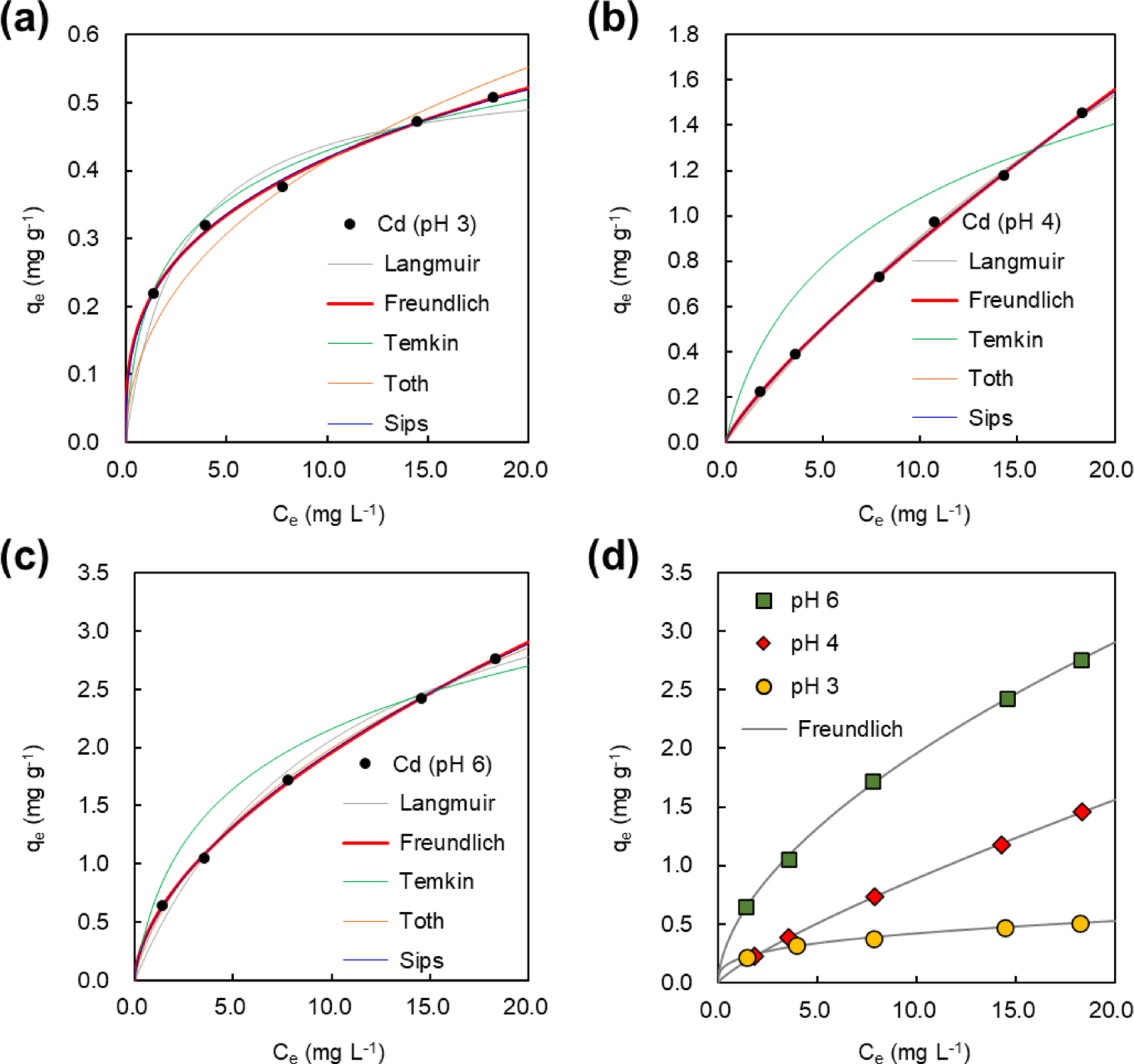
Cd(II) adsorption isotherms on PSC-3 and the corresponding isotherm model fits at different solution pH values. **a** pH 3, **b** pH 4, and **c** pH 6. **d** Comparison of the corresponding Freundlich isotherm model fits at different solution pH values

In aqueous media, cadmium predominantly exists as Cd2⁺; its uptake by activated carbon is commonly attributed to electrostatic interactions and ion-exchange/complexation processes involving oxygen-containing surface functionalities (e.g., –OH and –COOH) (Kumar et al. 2014). Because solution pH governs the speciation of metal ions and the protonation state of surface functional groups, it critically influences adsorption performance. To assess Cd(II) uptake by PSC-3, isotherm experiments were conducted at pH 3, 4, and 6; the equilibrium data were fit using several adsorption models. As summarized in Table 5 and Fig. 7, the Freundlich isotherm provided the best fit (highest R2) across all tested conditions, with ${n}_{F}$ values of 3.108, 1.232, and 1.755 at pH 3, 4, and 6, respectively. The fact that ${n}_{F}$ exceeded unity in all cases indicated favorable adsorption and supported a Freundlich-type behavior, implying the occurrence of adsorption on energetically heterogeneous sites rather than ideal monolayer saturation.

**Table 5:** Adsorption isotherm parameters for Cd(II) adsorption onto PSC-3

Model	Parameter	pH 3	pH 4	pH 6
Langmuir	${q}_{m}$ (mg g^−1^)	0.5549	4.8163	4.2552
	*b* (L mg^−1^)	0.3727	0.0232	0.0944
	${R}^{2}$	0.9465	0.9979	0.9889
Freundlich	${K}_{F}$ (L mg^−1^)	0.1991	0.1370	0.5274
	${n}_{F}$	3.1085	1.2320	1.7551
	${R}^{2}$	0.9963	0.9999	0.9995
Temkin	*B*	0.1110	0.5225	0.8239
	${K}_{T}$ (L mg^−1^)	4.6932	0.6871	1.2756
	${R}^{2}$	0.9836	0.9441	0.9622
Toth	${q}_{m}$ (mg g^−1^)	35.4977	13.8821	31.6234
	${n}_{T}$	0.1240	0.5983	0.2853
	${K}_{T}$ (L mg^−1^)	1.1749	0.0093	0.0527
	${R}^{2}$	0.9068	0.9989	0.9985
Sips	${q}_{m}$ (mg g^−1^)	2.8095	19.2935	23.0169
	${n}_{S}$	2.7021	1.1786	1.6235
	${K}_{S}$ (L mg^−1^)	0.0748	0.0069	0.0227
	${R}^{2}$	0.9959	0.9997	0.9995

Experimental conditions: initial Cd(II) concentration (C₀) = 2–20 mg L^−1^, adsorbent dosage = 0.15 g in 0.25 L of solution, solution pH = 3.0, 4.0, and 6.0, temperature = 25 °C, and equilibrium contact time = 1,440 min

**Table 6:** Adsorption kinetic parameters for Cd(II) adsorption onto PSC-3 in a binary-component system

Model	Parameter	PSC-3 (Binary)
Pseudo-first-order	${q}_{e}$ (mg g^−1^)	0.53
	${k}_{1}$ (min^−1^)	0.015
	${R}^{2}$	0.967
Pseudo-second-order	${q}_{e}$ (mg g^−1^)	0.58
	${k}_{2}$ (g mg^−1^ min^−1^)	0.037
	${R}^{2}$	0.997
Weber–Morris	${K}_{i}$ (mg g^−1^ min^−0.5^)	0.02
	${R}^{2}$	0.389
Urano–Tachikawa	${q}_{e}$ (mg g^−1^)	0.43
	${D}_{i}$ (m^2^ s^−1^)	3.097
	${R}^{2}$	3.09 × 10^–13^
S-shape	${q}_{e}$ (mg g^−1^)	0.60
	*n*	1.119
	*b* (min^−1^)	0.030
	${R}^{2}$	0.999

Experimental conditions: initial methylene blue concentration = 100 mg L^−1^, initial Cd(II) concentration (C₀) = 2.0 mg L^−1^, adsorbent dosage = 0.15 g in 0.25 L of solution, solution pH = 6.0, temperature = 25 °C, and contact time = 1,440 min

Notably, the pH_PZC of PSC-3 (6.88) suggests that the surface is positively charged over the investigated pH range (pH 3–6), which inhibits the purely electrostatic attraction of Cd(II). Therefore, to achieve a favorable Cd(II) uptake under these conditions, a substantial contribution from specific interactions, such as surface complexation and ion exchange at oxygen-containing functional groups, is required in addition to electrostatic effects. The variation in ${n}_{F}$ with pH further suggests that adsorption intensity and surface affinity are pH-dependent, likely reflecting changes in surface protonation and the availability and strength of binding sites.

The lowest Freundlich adsorption intensity parameter ($n_{F}$ = 1.232) was observed at pH 4. Because the solution pH was significantly lower than the pH_PZC of PSC-3 (6.88), a substantial fraction of oxygen-containing functional groups remained protonated, thereby reducing the availability of negatively charged adsorption sites for Cd(II) binding. Furthermore, competition between H^+^ ions and Cd(II) for surface complexation and ion-exchange sites likely suppressed adsorption intensity.

Although the $n_{F}$ value at pH 3 (3.108) was higher than that at pH 4, the Freundlich parameter is an empirical fitting parameter obtained through non-linear regression and may be affected by surface heterogeneity and fitting uncertainty. Therefore, the observed variation should not be interpreted as a strictly monotonic pH dependence. Importantly, all $n_{F}$ values exceeded unity, indicating favorable Cd(II) adsorption on PSC-3 throughout the investigated pH range. Overall, these results indicate that Cd(II) removal by PSC-3 proceeds via multi-site adsorption on a heterogeneous surface, with oxygen-containing functionalities serving as key binding and ion-exchange centers throughout the tested pH range.

#### Comparison of Cd(II) adsorption in single- and binary-component systems

**Fig. 8:**
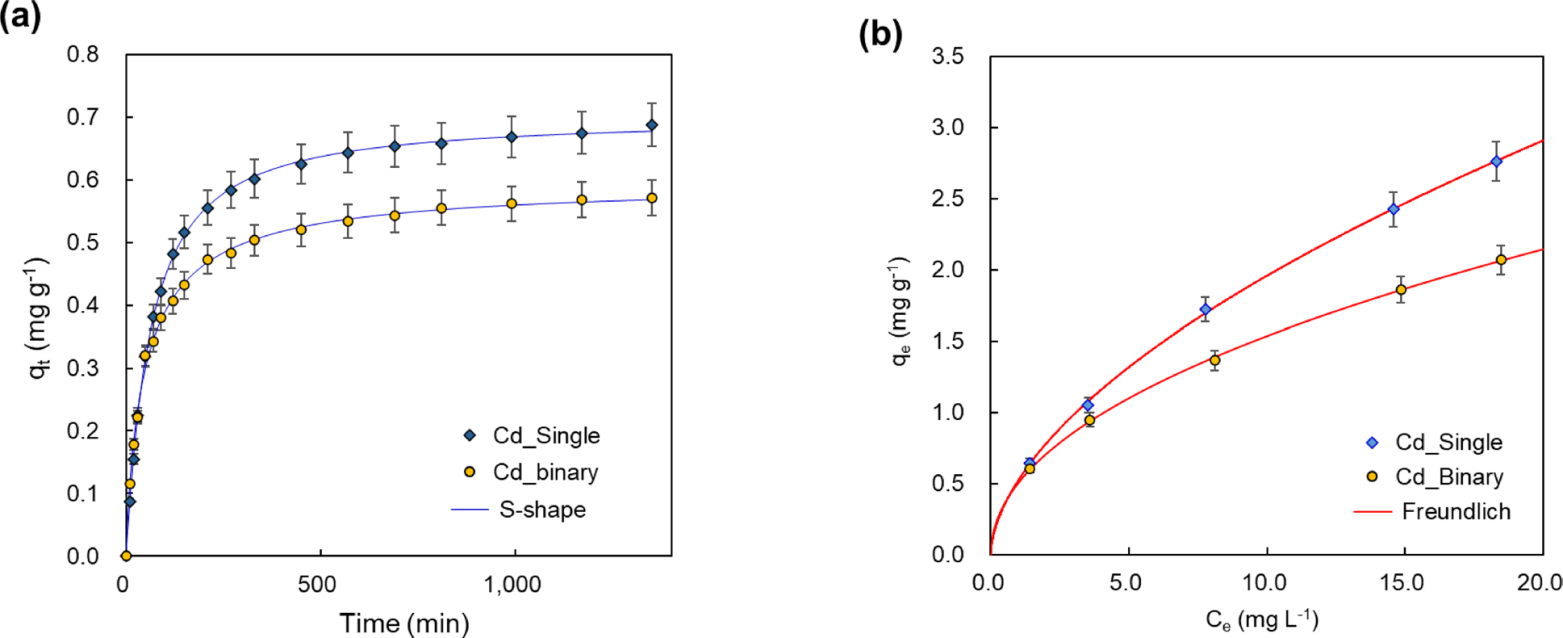
Comparison of Cd(II) adsorption in single- and binary-component systems. **a** Adsorption kinetics and, **b** adsorption isotherms of Cd(II) in single-component and binary-component systems

To evaluate the effect of competitive adsorption, the adsorption behavior of Cd(II) in single- and binary-component systems was compared using the kinetic and equilibrium isotherm results (Figs. 6, 7 and 8 and Tables 4, 5, 6 and 7). As shown in Figs. 6 and 8a, the adsorption kinetics of Cd(II) in both systems exhibited a similar trend, characterized by a rapid initial uptake followed by gradual attainment of equilibrium. In both systems, the S-shape kinetic model provided the best fit to the experimental data (R^2^ = 0.999), whereas the pseudo-second-order model also showed excellent agreement (R^2^ = 0.998 and 0.997 for the single- and binary-component systems, respectively). These results indicated that the overall adsorption kinetics and rate-controlling mechanisms were not significantly affected by the presence of MB.

**Table 7:** Adsorption isotherm parameters for Cd(II) adsorption onto PSC-3 in a binary-component system

Model	Parameter	PSC-3 (binary)
Langmuir	${q}_{m}$ (mg g^−1^)	2.72
	*b* (L mg^−1^)	0.149
	${R}^{2}$	0.9740
Freundlich	${K}_{F}$ (L mg^−1^)	0.51
	${n}_{F}$	2.082
	${R}^{2}$	0.9995
Temkin	*B*	0.57
	${K}_{T}$ (L mg^−1^)	1.74
	${R}^{2}$	0.9690
Toth	${q}_{m}$ (mg g^−1^)	18.45
	${n}_{T}$	0.25
	${K}_{T}$ (L mg^−1^)	0.21
	${R}^{2}$	0.9970
Sips	${q}_{m}$ (mg g^−1^)	17.53
	${n}_{S}$	1.93
	${K}_{S}$ (L mg^−1^)	0.03
	${R}^{2}$	0.9990

Experimental conditions: initial methylene blue concentration = 100 mg L^−1^, initial Cd(II) concentration (C₀) = 2–20 mg L^−1^, adsorbent dosage = 0.15 g in 0.25 L of solution, solution pH = 6.0, temperature = 25 °C, and equilibrium contact time = 720 min

The equilibrium adsorption behavior of Cd(II) also showed similar characteristics under single- and binary-component conditions (Fig. 8b and Tables 5–7). Compared with the single-component system at pH 6.0, Cd(II) adsorption in the binary-component system was slightly lower, indicating limited competition between MB molecules and Cd(II) ions for the available adsorption sites on PSC-3. Nevertheless, the Freundlich and Sips isotherm models provided excellent fits to the experimental data, suggesting that heterogeneous adsorption remained the dominant adsorption mechanism in both systems. Overall, the equilibrium adsorption behavior was not significantly altered by the presence of MB.

These findings demonstrated that PSC-3 maintained high adsorption performance under binary-component conditions despite the presence of competitive adsorption, highlighting its potential for the simultaneous removal of organic dyes and heavy metal ions from mixed wastewater.

### Simultaneous removal of MB and Cd(II) in a binary system

To evaluate the simultaneous removal of MB and Cd(II) by PSC-3 in a binary system, batch adsorption experiments were conducted at different pH values using an aqueous solution containing 50 mg L^−1^ MB and 2 mg L^−1^ Cd(II). The PSC-3 dosage was varied from 2 to 5 g L^−^1; the adsorption experiments were performed at 25 °C for 30 min. Under a representative binary condition (2 g L^−1^ PSC-3, pH 7, 25 °C, 30 min), the adsorption capacities of PSC-3 for MB and Cd(II) were 26.47 mg g^−1^ and 0.73 mg g^−1^, respectively.

**Fig. 9:**
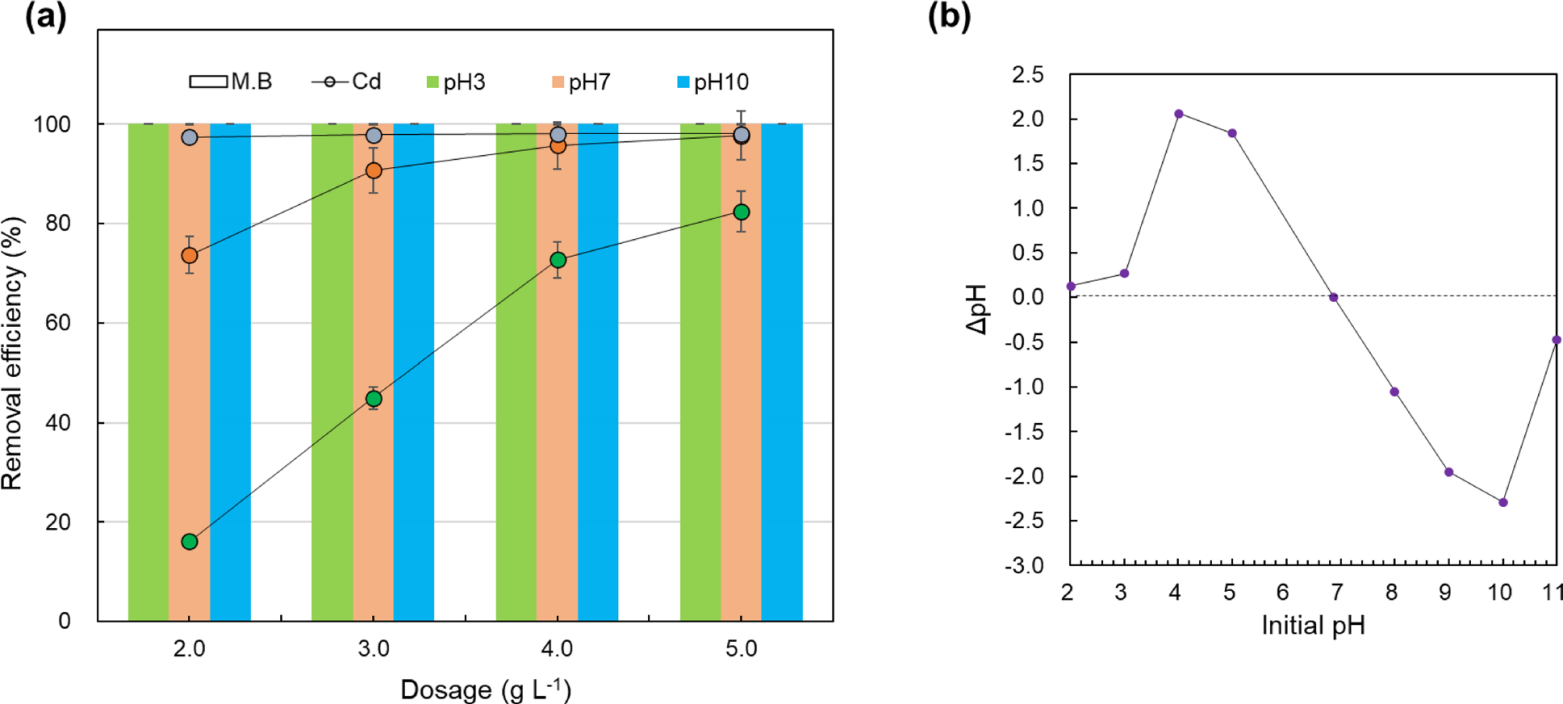
pH-dependent binary removal performance and pH-drift behavior of PSC-3. **a** Simultaneous removal of MB and Cd(II) by PSC-3 in a binary-component system at different solution pH values. Bars represent MB removal efficiency, whereas symbols represent Cd(II) removal efficiency. **b** ΔpH variation of PSC-3 as a function of the initial solution pH (pH-drift method)

As shown in Fig. 9a, PSC-3 achieved a nearly complete removal of MB (approximately 100%) over the entire investigated pH range, indicating a strong affinity of MB for PSC-3. This excellent adsorption performance is mainly attributed to pore filling and π–π interactions between MB molecules and the graphitized carbon framework of PSC-3.

In contrast, the removal efficiency of Cd(II) was strongly dependent on solution pH and increased markedly from acidic to neutral and alkaline conditions. Furthermore, increasing the PSC-3 dosage from 2 to 5 g L^−1^ consistently enhanced Cd(II) removal because of the improved availability of adsorption and ion-exchange sites.

The simultaneous removal performance of PSC-3 was further evaluated by comparing the adsorption behavior in single-component and binary-component systems. As summarized in Table 8 and Fig. 9a, the removal efficiency of MB remained essentially unchanged in the presence of Cd(II), with removal efficiencies consistently exceeding 99% under all investigated experimental conditions. In contrast, Cd(II) removal was slightly lower in the binary-component system than in the corresponding single-component system, indicating limited competitive adsorption between MB molecules and Cd(II) ions for the available adsorption sites on PSC-3. Nevertheless, PSC-3 maintained excellent removal efficiencies for both contaminants, demonstrating its promising applicability for the simultaneous treatment of wastewater containing organic dyes and heavy metal ions.

**Table 8:** Removal efficiencies of MB and Cd(II) in single- and binary-component systems at different adsorbent dosages

Adsorbate	Dosage (g L^−1^)	Removal efficiency (%)					
		Single-component system			Binary-component system		
		pH 3	pH 7	pH 10	pH 3	pH 7	pH 10
MB	2	99.8	99.9	99.9	99.9	99.9	99.9
	3	99.9	99.9	99.9	99.9	99.9	99.9
	4	99.8	99.9	100.0	99.9	100.0	99.9
	5	99.9	100.0	100.0	99.9	100.0	99.9
Cd(II)	2	18.8	77.6	98.6	16.2	73.7	97.4
	3	52.3	98.3	99.3	45.0	90.7	97.9
	4	81.6	99.5	99.9	72.7	95.7	98.1
	5	91.3	99.5	99.9	82.5	97.7	98.0

Experimental conditions: initial MB concentration = 50 mg L^−1^, initial Cd(II) concentration (C₀) = 2.0 mg L^−1^, adsorbent dosage = 2–5 g L^−1^, solution pH = 3.0, 7.0, and 10.0, temperature = 25 °C, and contact time = 30 min

Considering the pH_PZC of PSC-3 (6.88) and the results of the pH-drift method (Fig. 9b), the pronounced enhancement of Cd(II) removal above pH 7 is attributed to deprotonation of oxygen-containing surface functional groups, resulting in negatively charged adsorption sites that promote electrostatic attraction, ion exchange, and surface complexation. To distinguish adsorption from precipitation, an additional control experiment was performed at pH 10 in the absence of PSC-3. The control experiment confirmed that Cd(OH)_2_ precipitation was the dominant removal mechanism under strongly alkaline conditions. Therefore, the near-complete Cd(II) removal observed at pH 10 cannot be attributed exclusively to adsorption by PSC-3. Accordingly, the adsorption behavior discussed in this study primarily reflects the mechanisms operating under acidic and near-neutral conditions, whereas precipitation contributes significantly to Cd(II) removal at pH 10.

To further evaluate the adsorption performance of PSC-3, its adsorption capacities were compared with those of representative biomass-derived activated carbons reported in recent literature (Table 9). As summarized in Table 9, PSC-3 exhibited an MB adsorption capacity of 529.5 mg g^−1^, which is comparable to or higher than those reported for many biomass-derived activated carbons. Although the Cd(II) adsorption capacity (4.26 mg g^−1^) was comparable to those of several previously reported biomass-derived adsorbents, we further evaluated the simultaneous removal of MB and Cd(II) under binary-component conditions in the present study. This additional investigation provides valuable insights into the practical applicability of PSC-3 for the treatment of wastewater containing organic dyes and heavy metal ions.

**Table 9:** Comparison of adsorption capacities of representative biomass-derived activated carbons for methylene blue (MB) and Cd(II)

Precursor Material	Specific surface area (m^2^ g^−1^)	Adsorbent	Capacity (mg g^−1^)	References
Rubber seed pericarp	1245.7	MB	348	Jawad et al. (2022b)
Oil palm frond and palm kernel shell	–	MB	332	Jasri et al. (2023)
Sunflower pith	2090.0	MB	581	Baysal et al. (2018)
Chickpea peel	917.0	MB	200	Jahan et al. (2023)
Dragon fruit peels	756.3	MB	195.2	Jawad et al. (2021)
Coconut shell	873.5	MB	623.37	Tu et al. (2021)
Cassava peel	–	MB	286.41	Belcaid et al. (2024)
Pecan shell	2857.0	MB	529.50	This work
Rice husk	–	Cd(II)	8.58	Kumar and Bandyopadhyay (2006)
Rice husk ash	–	Cd(II)	3.04	Sharma et al. (2006)
Apple peels	–	Cd(II)	0.80	Abdolali et al. (2016)
Wheat bran	403.0	Cd(II)	2.56	Singh et al. (2006)
Garlic waste	–	Cd(II)	2.30	Negi et al. (2012)
Pecan shell	2857.0	Cd(II)	4.26	This work

### Adsorption mechanism

SEM observations and BET analysis revealed that PSC-3 possessed a well-developed porous structure with a high specific surface area (2857 m^2^ g^−1^), providing abundant adsorption sites for MB molecules and Cd(II) ions. The average pore diameter of PSC-3 was 2.25 nm, which is substantially larger than the molecular size of MB (approximately 0.84 nm), allowing MB molecules to readily diffuse into the internal pore network (Guarín et al. 2018). Therefore, the hierarchical porous structure of PSC-3 promotes rapid adsorbate diffusion and enhances the overall adsorption capacity.

FT-IR analysis further confirmed the presence of oxygen-containing functional groups, including hydroxyl (–OH), carbonyl (C=O), and carboxyl (–COOH) groups. These functional groups play important roles in Cd(II) adsorption through electrostatic attraction, ion exchange, and surface complexation. Furthermore, the pH_PZC value of 6.88 indicates that the PSC-3 surface becomes negatively charged under neutral and alkaline conditions, thereby favoring the adsorption of cationic metal ions.

**Fig. 10:**
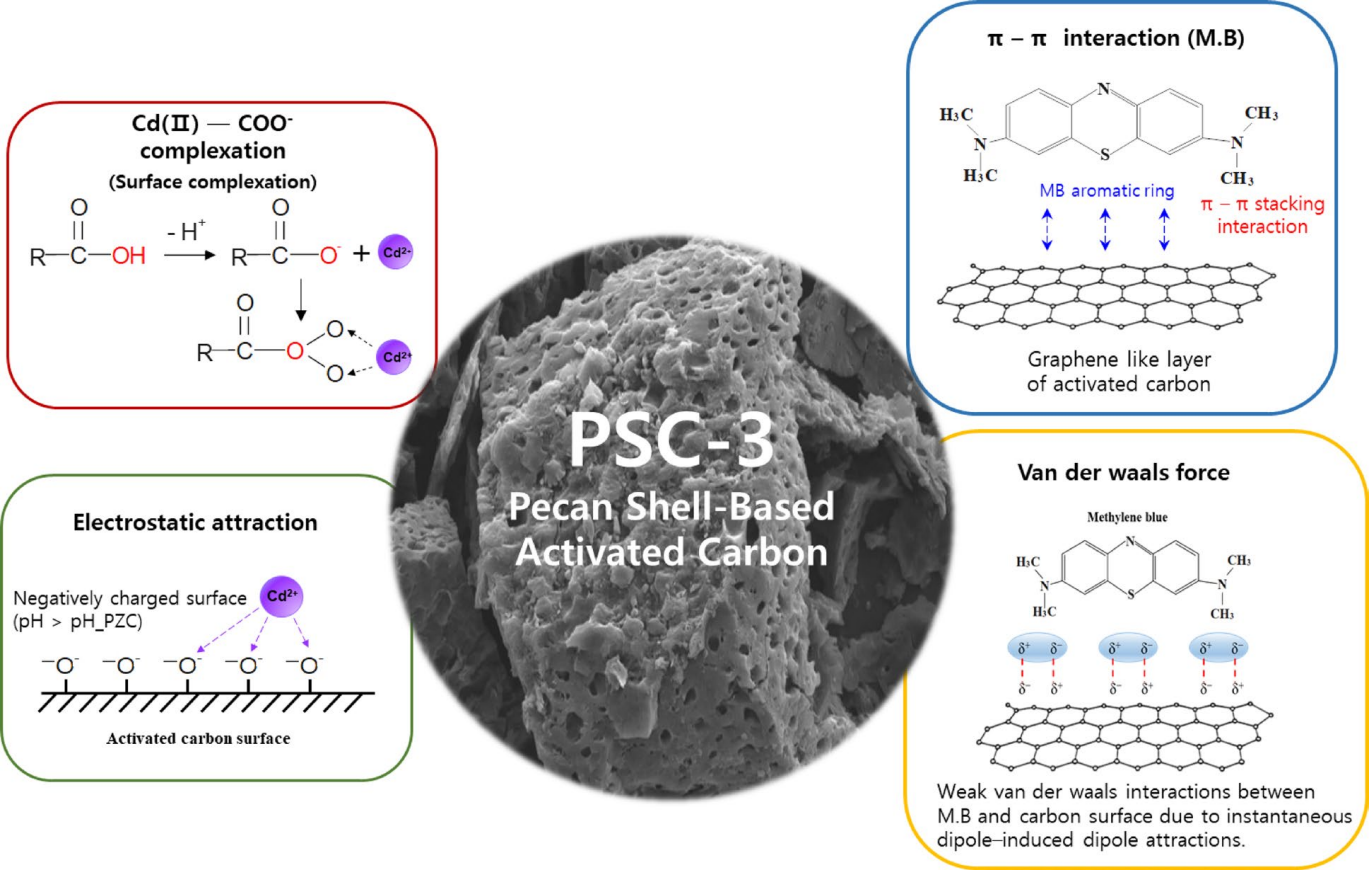
Proposed adsorption mechanism of methylene blue (MB) and Cd(II) on PSC-3

As illustrated in Fig. 10, MB adsorption mainly occurs through pore filling, π–π stacking interactions, and weak van der Waals interactions. The graphitized carbon framework of PSC-3 provides graphene-like aromatic domains that strongly interact with the aromatic rings of MB through π–π stacking. Additionally, electron-donating surface functional groups (e.g., –OH) increase the π–electron density of the carbon surface, thereby strengthening the π–π interactions and contributing to the rapid adsorption kinetics and high adsorption capacity of MB (Li et al. 2019; Surip et al. 2020).

In contrast, Cd(II) adsorption is dominated by electrostatic attraction and surface complexation with oxygen-containing functional groups. At solution pH values above the pH_PZC, surface carboxyl groups (–COOH) are deprotonated to form negatively charged carboxylate groups (–COO^−^), which electrostatically attract Cd(II) ions and facilitate surface complexation. Furthermore, Cd(II) ions form inner-sphere complexes with deprotonated carboxyl groups (–COO^−^), resulting in stable chemical adsorption.

Under binary-component conditions, MB molecules and Cd(II) ions compete for the available adsorption sites on PSC-3, resulting in a slight decrease in Cd(II) adsorption compared with the corresponding single-component system. This observation suggests limited competitive adsorption between MB molecules and Cd(II) ions. Under strongly alkaline conditions (pH 10), the additional filtration-only control experiment confirmed that Cd(OH)₂ precipitation became the dominant removal pathway. Therefore, the near-complete Cd(II) removal observed at pH 10 cannot be attributed exclusively to adsorption by PSC-3, whereas electrostatic attraction and surface complexation are the dominant adsorption mechanisms under acidic and near-neutral conditions.

Overall, the superior adsorption performance of PSC-3 can be attributed to the synergistic effects of its hierarchical porous structure, high specific surface area, oxygen-containing surface functional groups, and graphitized carbon domains. These characteristics collectively promote pore filling, π–π stacking interactions, electrostatic attraction, surface complexation, and limited competitive adsorption, enabling the efficient simultaneous removal of methylene blue and Cd(II) from aqueous solutions.

### Regeneration and reusability of PSC-3

**Fig. 11:**
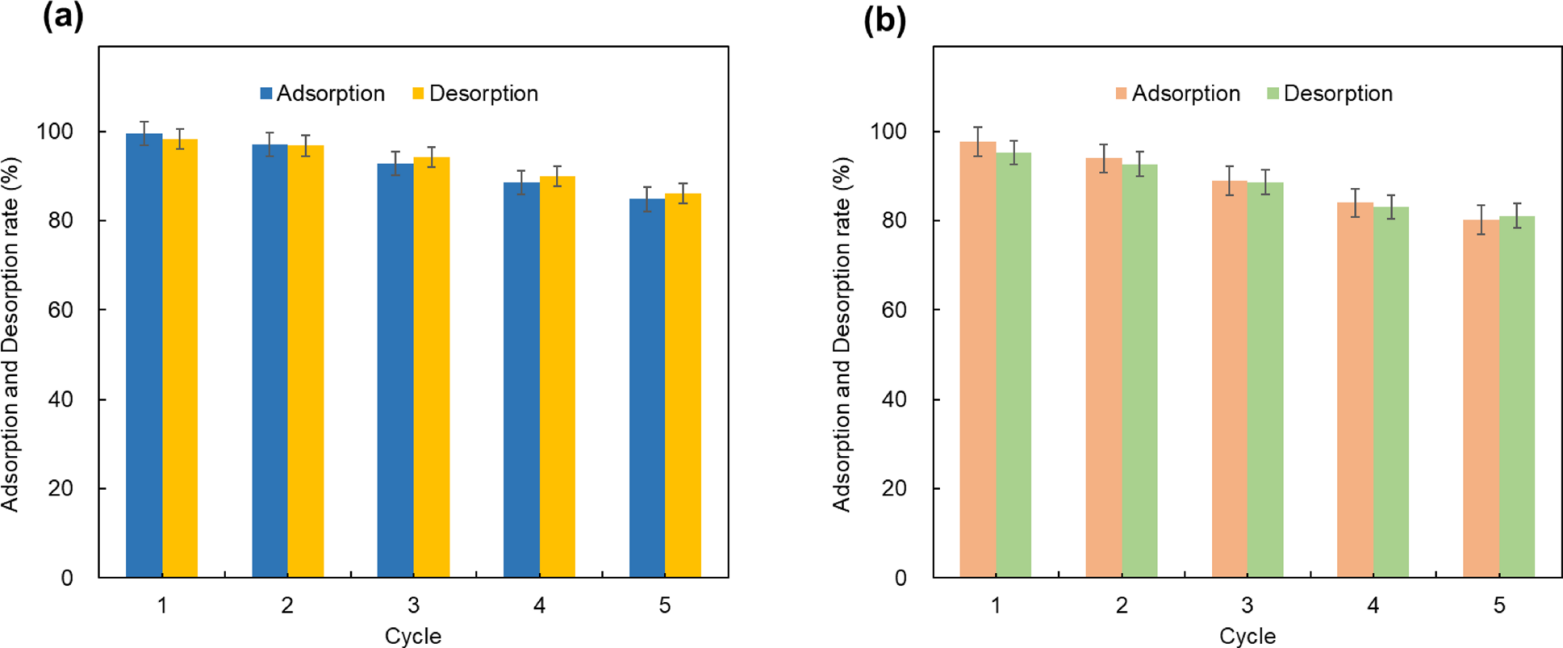
Adsorption–desorption regeneration performance of PSC-3 over five consecutive cycles. **a** MB and **b** Cd(II). Adsorption–desorption regeneration over five consecutive cycles. Ethanol (90%, v/v) and 0.1 M HCl were used as desorption agents for MB and Cd(II), respectively

The regeneration performance of PSC-3 was evaluated over five consecutive adsorption–desorption cycles using 90% (v/v) ethanol for MB and 0.1 M HCl for Cd(II) as desorption agents (Fig. 11). For both contaminants, the adsorption efficiency gradually decreased with increasing regeneration cycles, which can be attributed to incomplete desorption and the partial loss of active adsorption sites. Nevertheless, PSC-3 retained approximately 85% of its initial adsorption efficiency for MB and 80% for Cd(II) after five regeneration cycles, demonstrating good regeneration capability and structural stability. These results indicate that PSC-3 is a promising reusable adsorbent for the treatment of wastewater containing organic dyes and heavy metal ions.

### Economic feasibility of PSC-3

The economic feasibility of PSC-3 was evaluated based on the estimated production cost of the adsorbent. The production cost was calculated considering the consumption of KOH, electricity required for carbonization and activation, washing water, acid washing, and drying processes. Because pecan shells are an agricultural by-product, the raw material cost was assumed to be negligible.

Supplementary Table S1 summarizes the estimated laboratory-scale production cost of PSC-3 and compares it with that of representative commercial activated carbon. Supplementary Table S1a presents the detailed production cost calculations for PSC-3 preparation. Additionally, the production cost per gram of methylene blue removed was estimated using the Langmuir adsorption capacity and the production cost of the adsorbent.

Based on a laboratory-scale batch using 35 g of pecan shell biomass and 105 g of KOH, the total production cost of PSC-3 was estimated to be approximately 1968 KRW per batch. Considering a final PSC-3 yield of 12 g, the production cost was calculated to be 164 KRW g^−1^, corresponding to approximately 0.119 USD g^−1^ (119 USD kg^−1^).

Although the laboratory-scale production cost of PSC-3 is currently higher than the market price of conventional activated carbon, PSC-3 exhibits a high adsorption capacity (Langmuir Qₘₐₓ = 529.5 mg g^−1^) and is produced from an abundant agricultural waste biomass. Therefore, the cost-effectiveness of PSC-3 should be evaluated in terms of production cost, adsorption performance, and sustainability. Moreover, the production cost is expected to decrease substantially through process optimization, reduced chemical consumption, and large-scale continuous production.

## Conclusions

In this study, pecan shell–based activated carbon (PSBAC) was successfully prepared via KOH activation and pyrolysis; its adsorption performance toward methylene blue (MB) and Cd(II) was systematically evaluated. KOH activation markedly enhanced pore development and increased the specific surface area; the optimized sample (PSC-3) exhibited a well-developed porous structure and excellent adsorption performance. Kinetic and equilibrium analyses showed that MB adsorption was well described by the pseudo-second-order kinetic model and Freundlich isotherm model, whereas the adsorption behavior of Cd(II) was adequately described by the pseudo-second-order kinetic model and Freundlich and Sips isotherm models. In the binary-component system containing MB and Cd(II), PSC-3 achieved nearly complete MB removal over a wide pH range, whereas Cd(II) removal was strongly dependent on solution pH and significantly improved under neutral-to-alkaline conditions, consistent with the pH_PZC of PSC-3 (6.88). These results indicated that the deprotonation of oxygen-containing surface functional groups promoted Cd(II) adsorption through electrostatic attraction, ion exchange, and surface complexation, whereas hydrolysis/precipitation additionally contributed under strongly alkaline conditions. Overall, these findings demonstrate that pecan shells are a cost-effective and sustainable precursor for producing high-performance activated carbon, with strong potential for the simultaneous removal of organic dyes and heavy metal ions from mixed wastewater.

Future studies should focus on the regeneration and long-term stability of PSC-3, optimization of continuous-flow adsorption systems, and pilot-scale evaluation using real industrial wastewater containing multiple organic and inorganic contaminants. Additionally, adsorption thermodynamics (ΔG°, ΔH°, and ΔS°) should be investigated over a range of temperatures to further elucidate the spontaneity and energetic characteristics of the adsorption process. These studies will provide a superiorly comprehensive understanding of the adsorption mechanism and facilitate the practical application of pecan shell–derived activated carbon for sustainable wastewater treatment and biomass valorization.

## Supplementary Information

Below is the link to the electronic supplementary material.

Supplementary Material 1

## Data Availability

Data supporting the findings of this study are available from the corresponding author upon reasonable request.
