# Supplementary material for: Simultaneous removal of methylene blue and Cd(II) by pecan shell–based activated carbon: adsorption kinetics, isotherms, and physicochemical characterization
Source: Bioresour Bioprocess. 2026 Aug 17;13(1):118. doi: 10.1186/s40643-026-01117-1 (PMC13481940; doi:10.1186/s40643-026-01117-1)
Supplement: Supplementary file 1 [file 40643_2026_1117_MOESM1_ESM.docx]

**Supplementary Tables**

**Supplementary Table S1.** Estimated production cost and economic comparison of PSC-3 with representative commercial activated carbon.

| Parameter | PSC-3 | Commercial activated carbon |
| --- | --- | --- |
| Raw material | Pecan shell waste | Coal/coconut shell |
| BET surface area (m² g⁻¹) | 2857 | 800–1500 |
| Production cost (USD g⁻¹) | 0.119 | 0.005 |
| Production cost (USD kg⁻¹) | 119 | 5.0ᵃ |
| MB adsorption capacity, Langmuir Qmax (mg g⁻¹) | 529.50 | ~~-~~ |
| Cost per gram of MB removed (USD g⁻¹ MB) | 0.225 | ~~-~~ |

ᵃ Representative market price of industrial-grade activated carbon used as a benchmark for economic comparison.

**Supplementary Table S2.** Estimated production cost for preparation of PSC-3.

| Description of item required  For PSC-3 preparation | Unit cost | No. of units | Cost (KRW) |
| --- | --- | --- | --- |
| Pecan shell biomass | 0 | 35 g | 0 |
| KOH | 6,000 KRW kg⁻¹ | 0.105 kg | 630 |
| Electricity for carbonization | 134 KRW kWh⁻¹ | 2.5 kWh | 335 |
| Electricity for activation | 134 KRW kWh⁻¹ | 3.75 kWh | 503 |
| Washing water | Estimated | - | 200 |
| HCl washing solution | Estimated | - | 300 |
| Total production cost (per batch) |  |  | 1,968 |
| Final PSC-3 yield (per batch) |  | 12 g |  |
| Estimated production cost of PSC-3 |  |  | 164 KRW g⁻¹ (0.119 USD g⁻¹; 119 USD kg⁻¹) |

Based on a final PSC-3 yield of 12 g per batch, the estimated laboratory-scale production cost of PSC-3 was calculated to be 164 KRW g⁻¹, corresponding to approximately 0.119 USD g⁻¹ (119 USD kg⁻¹).
